# An Interesting Case of Pregnancy

**Published:** 1861-12

**Authors:** M. M. Eaton

**Affiliations:** Peoria


### AN INTERESTING CASE OF PREGNANCY.

By M. M. EATON, M. D., Peoria.

Was called to visit Mrs. S. at her residence, No. — ——— street, October 13th, 1861. Found her in bed, looking anemic, pulse 100, and complaining of flooding, and pains like those of labor, saying, however, that she was not in the family way, as she had been flooding almost constantly, a little all summer. I requested an examination, which was granted; and on making the same, I detected blood flowing very freely *per vaginam;* the bed clothes were completely saturated and a pool was standing by her hips. The *os uteri* was dilated to the size of the quarter of a dollar, and through this an inferior member of a fœtus protruded. I made slight traction on it, and soon brought away a dead fœtus, apparently about four months, somewhat shriveled, but well formed. The funis was broken, but did not bleed. The placenta being retained, and hæmorrhage still going on, I gave the *Vinum Ergota* to bring on uterine contractions.

After the expulsion of the "after birth," no hæmorrhage of account occurred. I found half the placenta shriveled up and of a dark brown color, the remainder as well as the cord so soft that I could easily tear them to pieces with my fingers. There was however no offensive odor emitted.

The fœtus I have in my museum. After my patient had revived, I learned the following history of her case, which some days after she corroborated.

She is of German descent, is 47 years old, has had a large family, the youngest child being 5 years old. She has always been healthy. Was regular from the time she weaned her last babe till the last of December, 1860, when her catamenia ceased, and for four months she had morning sickness, after which her menstrua returned, and had continued nearly daily till I was called. She said she had been getting weak all summer, but supposing it was the "turn of life" which caused her irregularities, she did not consult me. The above is also her husband's story.

I have reported this case, thinking that she conceived last Fall, when her menstruation ceased; that gestation went on naturally four mounths, when a partial separation of the placenta took place, causing the flowing during the summer, which she thought to be her courses; that this being the case, the fœtus ceased to grow, owing to deficient nutrition, and remained in *statu quo* till the Fall turn, when labor set in, resulting as above stated. The views of Professors Brainard, Allen or Miller requested.

